# Development of a predictive score for hypothermia risk during continuous kidney replacement therapy in critically ill patients

**DOI:** 10.1080/0886022X.2026.2676486

**Published:** 2026-06-01

**Authors:** Panisara Chummano, Thongkran Thongkam, Naphat Chansatchanali, Yutthasat Sonprasom, Wachiranun Sirikul, Konlawij Trongtrakul

**Affiliations:** a Faculty of Medicine, Chiang Mai University, Chiang Mai, Thailand; b Department of Community Medicine, Faculty of Medicine, Chiang Mai University, Chiang Mai, Thailand; c Division of Pulmonary, Critical Care, and Allergy, Department of Internal Medicine, Faculty of Medicine, Chiang Mai University, Chiang Mai, Thailand

**Keywords:** Hypothermia, renal replacement therapy, critically illness, risk assessment, models, statistical

## Abstract

**Backgroud:** Hypothermia is a frequent adverse event during continuous kidney replacement therapy (CKRT) and is associated with clinically relevant complications. However, evidence regarding its predictors remains limited. We aimed to develop a clinical prediction score to facilitate early risk stratification and targeted preventive management.

**Methods:** We conducted a retrospective cohort study of critically ill adults receiving CKRT in the Medical Intensive Care Unit, Faculty of Medicine, Chiang Mai University, Thailand, between February 2018 and August 2024. Hypothermia was defined as body temperature <35 °C occurring within 48 h after CKRT initiation. Multivariable logistic regression was utilized to identify independent predictors, and a prediction score was derived from the regression coefficients.

**Results:** Among 214 cases (mean age 65 years; 38.3% female), hypothermia occurred in 51.9% (*n* = 111 cases). Independent predictors included respiratory failure at ICU admission (+13 points), total bilirubin (+1 point per mg/dL), APACHE-II score (+1.5 points per APACHE-II point), pre-CKRT serum creatinine (- 2 points per mg/dL), and pre-CKRT body temperature (−1 point per 0.1 °C). The score exhibited good discriminative ability (C-statistic 0.78; 95% CI 0.71–0.83). A cutoff value of 20.0 yielded a sensitivity of 78% (95% CI 70–86), specificity of 62% (95% CI 52–72), positive likelihood ratio of 2.07 (95% CI 1.59–2.70) and negative likelihood ratio of 0.35 (95% CI 0.24–0.51).

**Conclusion:** Hypothermia is common during CKRT, and the proposed score may enable practical risk stratification and early identification of patients who could benefit from targeted preventive strategies.

## Introduction

Hypothermia is a common and clinically significant complication during continuous kidney replacement therapy (CKRT) in critically ill patients with renal failure. Reported incidence ranges from 23.7% to 53.0% [1–4], with mortality rates among affected patients reaching 58.2% to 71.0% [2–4]. The extracorporeal circulation inherent to CKRT, combined with the use of large volumes of dialysate or replacement fluids, contributes substantially to heat loss, particularly in hemodynamically unstable patients with impaired thermoregulation [3].

In patients requiring CKRT, thermoregulatory capacity may be impaired due to systemic inflammation, sepsis, hemodynamic instability, and uremia-related autonomic dysfunction. The development of hypothermia in this context may further aggravate physiological instability, contributing to an increased susceptibility to infection, shivering, metabolic stress, coagulopathy, arrhythmias, and vasopressor requirements [2,3,5–8]. Thus, hypothermia during CKRT may reflect the complexity and severity of critical illness in this population.

Although several studies have identified risk factors associated with hypothermia during CKRT [2–4], research specifically addressing risk prediction remains limited. The development of a clinically applicable prediction score may facilitate early risk stratification and enable timely preventive interventions. Therefore, this study aimed to develop and evaluate a clinical prediction score for hypothermia during CKRT in critically ill patients.

## Materials and methods

We conducted a retrospective cohort study of adult patients who received CKRT in the Medical Intensive Care Unit (ICU) at the Faculty of Medicine, Chiang Mai University, Chiang Mai, Thailand, between February 2018 and August 2024. This study protocol was approved by the Research Ethics Committee (REC) of Faculty of Medicine, Chiang Mai University, Chiang Mai, Thailand with approval number FAC-MED-2567-0182 on 28 March 2024. The study was performed in accordance with the Declaration of Helsinki, which outlines ethical principles for medical research involving human subjects. The requirement for informed consent was waived due to the retrospective nature of the study and minimal risks to participants.

### CKRT protocol

Patients were considered for CKRT based on established clinical indications, including severe acute renal failure, refractory electrolyte imbalances, volume overload, uremic complications, sepsis-associated acute kidney injury, or liver failure complicated by renal dysfunction.

CKRT was delivered using continuous venovenous hemofiltration (CVVH), continuous venovenous hemodialysis (CVVHD), or continuous venovenous hemodiafiltration (CVVHDF). The selection of CKRT modality, system, filter, prescribed effluent dose (25–40 mL/kg/hour), and anticoagulation strategy was determined by the attending nephrologist in accordance with standard institutional practice.

### Inclusion and exclusion criteria

Critically ill patients aged 18 years or older who received CKRT were eligible for inclusion. The analysis was restricted to data from the first 48 h following CKRT initiation. Patients were excluded if they underwent therapeutic hypothermia, had baseline hypothermia (body temperature <35 °C) prior to CKRT initiation, or experienced repeated CKRT episodes, defined as interruptions lasting more than 6 h followed by reinitiation within 48 h. In patients with repeated episodes, only the initial episode was included in the analysis.

### Data collection

Data collected consisted of patient demographic characteristics, including age, sex, body mass index (BMI), preexisting comorbidities, diagnosis at ICU admission, source of infections, vital signs, laboratory values, severity of illness at CKRT initiation, measured by the sequential organ failure assessment (SOFA) score, and the acute physiology and chronic health evaluation-II (APACHE-II) score.

Data on supportive therapy were recorded, including the use of mechanical ventilation, vasopressor and inotropic agents, and the calculated vasopressor inotropic score.

CKRT-related data included indication, modality, prescribed dose, fluid balance prior to CKRT initiation, and time from ICU admission to CKRT initiation.

### Definition

Hypothermia was defined as a body temperature <35 °C occurring within the initial 48 h of CKRT, consistent with previous literature [2]. This threshold was selected to represent a clinically meaningful degree of hypothermia during CKRT. Body temperature was measured *via* the axillary route using a standardized digital thermometer, with recordings obtained every 4 h as part of routine ICU monitoring. The baseline pre-CKRT body temperature was defined as the last recorded measurement prior to CKRT initiation.

### Outcomes

The outcome of interest for model development was hypothermia occurring within the first 48 h after CKRT initiation. ICU mortality, hospital mortality, ICU length of stay, hospital length of stay, CKRT duration, duration of vasopressor use, and duration of mechanical ventilation, were summarized descriptively and compared between groups in secondary analyses.

### Sample size calculation

Based on the study by Morsch et al. [2], the incidence of hypothermia during the first 48 h of CKRT initiation was 52.7%. Applying the rule of thumb of 10 events per predictor and assuming 10 predictors in the model [9], a minimum sample size of 192 cases was required. To account for an anticipated 10% rate of missing data, the final sample size was increased to 214 cases.

As a contemporary sensitivity analysis, we additionally applied the Riley framework under multiple plausible assumptions (10, 8, and 5 predictor parameters), assuming an anticipated C-statistic of 0.75 and target shrinkage of 0.90. The estimated minimum required sample size ranged from 384 to 434 patients, depending on assumed model complexity. Under the final model assumption (5 parameters), the shrinkage-based criterion for limiting overfitting was 217 patients, which was close to the available cohort size of 214 patients.

### Statistical analysis

Continuous variables were presented as mean ± standard deviation (SD) or median and interquartile range (IQR), as appropriate. Categorical variables were presented as numbers and percentages. Continuous variables were compared using the Student T-test or Mann-Whitney U test, as appropriate. Categorical variables were compared using Fisher’s exact test. All comparisons were analyzed by two-tailed tests, with *p* < 0.05 indicating statistical significance.

### Model development

A multivariable logistic regression model was used to develop the prediction score for hypothermia during CKRT. Candidate predictors were selected based on clinical relevance and entered into the model using backward elimination with a removal criterion of *p* < 0.10.

Multicollinearity was assessed using variance inflation factors (VIF), with VIF < 10 and tolerance > 0.20 considered acceptable.

A point-based prediction score was derived by scaling each regression coefficient relative to the smallest absolute coefficient in the final model, followed by rounding to the nearest integer or 0.5 increment. Subsequently, these individual component scores were then summed to obtain the total hypothermia prediction score. Model discrimination was evaluated using the concordance statistic (C-statistic) with corresponding 95% confidence interval (95% CI).

### Model calibration and validation

Model calibration between predicted risk and observed outcome was assessed graphically using calibration-in-the-large (CITL) and calibration plots, and statistically evaluated using the Hosmer-Lemeshow goodness-of-fit (HL-GOF) test. Internal validation was performed using bootstrapping with 1,000 replications to estimate model optimism.

### Optimal cutoff selection and risk stratification

The optimal cutoff value of the prediction score was determined using the Youden index. When adjacent cutoff values demonstrated comparable diagnostic performance, a clinically interpretable threshold was selected to enhance practical applicability. Sensitivity, specifically, positive likelihood ratio (LR+), and negative likelihood ratio (LR-), along with corresponding 95% CI, were calculated for each candidate cutoff.

For risk stratification, score strata boundaries were defined to reflect clinically meaningful score ranges while ensuring reasonable group sizes.

### Decision curve analysis for clinical utility

Decision curve analysis (DCA) was conducted to assess the clinical utility of the prediction score by estimating net benefit across a range of threshold probabilities, compared with treat all and treat none strategies.

### Post hoc sensitivity analysis

A *post hoc* sensitivity analysis was conducted to evaluate the robustness of the model after excluding patients with end-stage kidney disease (ESKD), given that baseline serum creatinine in this subgroup may not reflect acute physiological changes. The multivariable model was refitted using the same predictors, and model discrimination was reassessed using C-statistic and its 95% CI.

### Hypothermia-related ICU mortality

ICU mortality was evaluated using time-to-event analysis. Kaplan-Meier survival curves were constructed to compare ICU survival between patients with and without hypothermia, and differences were assessed using the log-rank test. Cox proportional hazards regression analysis was performed to estimate crude and adjusted hazard ratios for ICU mortality.

## Results

### Study population

A total of 214 patients were included. Hypothermia within the first 48 h after CKRT initiation occurred in 51.9% (*n* = 111), with a median time to onset of 7 h (IQR 4, 12). Baseline characteristics are summarized in Table 1. Compared with patients who did not develop hypothermia, those who developed hypothermia were older (median age 69 vs. 61 years, *p* = 0.002), had a lower BMI (22.2 kg/m^2^ vs. 23.7 kg/m^2^, *p* = 0.008), more frequently presented with respiratory failure at ICU admission (33.3% vs. 16.5%, *p* = 0.005), and had lower pre-CKRT body temperature (36.3 °C vs. 37.0 °C, *p* < 0.001). They also had lower serum creatinine levels before CKRT initiation (3.2 mg/dL vs. 4.2 mg/dL, *p* < 0.001), and higher APACHE-II scores (30 vs. 27, *p* < 0.001). Other baseline variables were not significantly different between groups.

**Table 1.** Baseline characteristics between hypothermia and without hypothermia.

Variables	Total (*n* = 214)	Hypothermia (*n* = 111)	No hypothermia (*n* = 103)	*p*-Value
Age (years)	65 ± 17	69 ± 16	61 ± 18	0.002
Female, *n* (%)	82 (38.3)	45 (40.5)	37 (35.9)	0.57
Body mass index (kg/m^2^)	22.9 ± 4.3	22.2 ± 3.5	23.7 ± 4.9	0.008
Preexisting Comorbidities, *n* (%)				
Hypertension	117 (54.7)	62 (55.9)	55 (53.4)	0.78
Diabetes mellitus	80 (37.4)	38 (34.2)	42 (40.8)	0.40
Chronic kidney disease	50 (23.4)	25 (22.5)	25 (24.3)	0.87
End stage kidney disease	29 (13.2)	11 (9.9)	18 (17.5)	0.12
Malignancy	47 (22.0)	24 (21.6)	23 (22.3)	>0.99
Dyslipidemia	44 (20.6)	22 (19.8)	22 (21.4)	0.90
Other comorbidities	126 (58.9)	66 (59.5)	60 (58.3)	0.89
Septic shock at ICU admission, *n* (%)	172 (80.4)	90 (81.1)	82 (79.6)	0.86
Source of infection, *n* (%)				
Respiratory tract	98 (45.8)	52 (46.9)	46 (44.7)	0.79
Urosepsis	36 (16.8)	16 (14.4)	20 (19.4)	0.36
Gastrointestinal tract	26 (12.2)	12 (10.8)	14 (13.6)	0.68
Others	40 (18.7)	22 (19.8)	18 (17.5)	0.73
Organ failure at ICU admission, *n* (%)				
Cardiovascular	25 (11.7)	11 (9.9)	14 (13.6)	0.52
Renal	24 (11.2)	10 (9.0)	14 (13.6)	0.39
Respiratory	54 (25.2)	37 (33.3)	17 (16.5)	0.005
Gastrointestinal	11 (5.1)	2 (1.8)	9 (8.7)	0.029
Others	17 (7.9)	6 (5.4)	11 (10.7)	0.21
Vital signs at CKRT Initiation				
Pre-CKRT Body temperature (°C)	36.6 ± 1.1	36.3 ± 0.9	37.0 ± 1.2	<0.001
Mean arterial pressure (mm Hg)	72 ± 12	72 ± 12	73 ± 12	0.58
Heart rate (bpm)	99 ± 26	95 ± 27	102 ± 25	0.044
Respiratory rate (bpm)	21 ± 6	22 ± 6	21 ± 5	0.06
Laboratory before CKRT Initiation				
Hemoglobin (g/dL)	8.5 ± 2.4	8.3 ± 2.2	8.7 ± 2.6	0.27
White blood cell count (10^3^ cells/mm^3^)*	13.9 (8.0, 20.1)	14.2 (7.2, 20.7)	13.5 (8.4, 19.9)	0.80
Platelet count (10^3^ cells/mm^3^)*	110 (57, 198)	111 (57, 205)	108 (52, 198)	0.82
Total bilirubin (mg/dL)*	1.8 (0.7, 5.2)	2.2 (0.8, 6.6)	1.4 (0.62, 4.3)	0.06
Baseline serum creatinine (mg/dL)*	1.2 (0.8, 2.4)	1.1 (0.8, 2.2)	1.3 (0.8, 3.1)	0.16
Serum creatinine (mg/dL)*	3.6 (2.4, 5.6)	3.2 (2.1, 4.5)	4.2 (2.7, 6.4)	<0.001
Bicarbonate (mEq/L)	15 ± 6	15 ± 6	15 ± 6	0.42
Serum lactate (mEq/L)	7.0 (2.7, 18.2)	7.3 (2.7, 18.5)	6.7 (2.5, 17.8)	0.72
SOFA score	13 ± 3	14 ± 3	13 ± 3	0.27
APACHE-II score	28 ± 6	30 ± 6	27 ± 6	<0.001
Management before CKRT initiation				
Vasopressor usage, *n* (%)	202 (94.4)	107 (96.4)	95 (92.2)	0.24
Vasopressor inotropic score*	48 (22, 90)	50 (24, 97)	44 (22, 87)	0.58
Mechanical ventilation, *n* (%)	208 (97.2)	108 (97.3)	100 (97.1)	>0.99
CKRT Information				
Time from ICU admission before CKRT initiation (hours)*	7 (3, 24)	7 (3,19)	9 (3, 27)	0.42
Fluid balance before CKRT (L) *	2.2 (0.8, 4.8)	2.1 (0.8, 5.4)	2.3 (0.8, 4.7)	0.81
Urine output before CKRT (mL) *	81 (3, 680)	75 (8, 700)	100 (0, 590)	0.72
CKRT Indication, *n* (%)				
Severe AKI	133 (62.2)	70 (63.1)	63 (61.2)	0.78
Electrolyte imbalance	75 (35.1)	33 (29.7)	42 (40.8)	0.12
Volume overload	14 (6.5)	11 (9.9)	3 (2.9)	0.05
Uremic complication	14 (6.5)	7 (6.3)	7 (6.8)	>0.99
Mode of CKRT initiation, *n* (%)				0.45
CVVH	45 (21.0)	26 (23.4)	19 (18.4)	
CVVHD	45 (21.0)	20 (18.0)	25 (24.3)	
CVVHDF	124 (58.0)	65 (58.6)	59 (57.3)	
Dose of CKRT initiation (mL/kg/h)	31.4 ± 5.6	31.3 ± 5.3	31.5 ± 5.8	0.76
Lowest body temperature during CKRT (°C)	34.8 ± 1.4	33.8 ± 1.0	36.0 ± 0.9	<0.001
Temperature variation (°C)*	1.6 (0.8, 2.8)	2.4 (1.6, 3.4)	1.0 (0.2, 1.6)	<0.001
Time to hypothermia onset (hours)	n/a	7 (4, 12)	n/a	n/a
Outcomes				
ICU mortality, *n* (%)	127 (59.4)	72 (64.9)	55 (53.4)	0.10
Hospital mortality, *n* (%)	143 (66.8)	78 (70.3)	65 (63.1)	0.31
ICU length of stay (days)*	4 (2, 9)	4 (2, 8)	5 (2, 10)	0.15
Hospital length of stay (days)*	10 (5, 19)	10 (4,19)	10 (5, 19)	0.60
CKRT duration (hours)*	28 (11, 52)	28 (12, 47)	28 (11, 55)	0.83
Vasopressor duration (hours)*	34 (17, 61)	38 (19, 61)	33 (15, 61)	0.71
Mechanical ventilator duration (days)*	4 (2, 9)	4 (2, 8)	4 (2, 10)	0.29

Continuous variables are reported as mean ± SD. * indicates median (IQR).

*Abbreviations:* APACHE-II score: acute physiology and chronic health evaluation II score; BMI: body mass index; CKRT: continuous kidney replacement therapy; ICU: intensive care unit; SOFA: sequential organ failure assessment

### Risk factors for hypothermia during CKRT

In univariable logistic regression analyses, age, BMI, respiratory failure at ICU admission, pre-CKRT body temperature, total bilirubin, serum creatinine, and APACHE-II score were associated with hypothermia during CKRT (all *p* < 0.10), as shown in Supplementary Table 1.

From multivariable model (Table 2), respiratory failure at ICU admission (OR 2.63; 95% CI 1.25–5.57; *p* = 0.011), higher total bilirubin (OR 1.08; 95% CI 1.02–1.14; *p* = 0.008), and higher APACHE-II score (OR 1.11; 95% CI 1.05–1.17; *p* < 0.001) were independently associated with an increased risk of hypothermia. Conversely, higher serum creatinine (OR 0.85; 95% CI 0.76–0.96; *p* = 0.006) and higher pre-CKRT body temperature (OR 0.92; 95% CI 0.89–0.96; *p* < 0.001) were associated with a lower likelihood of hypothermia.

**Table 2.** Derived prediction score for hypothermia during CKRT.

Predictors	OR (95% CI)	*p*-Value	Coefficient	Score
Respiratory failure at ICU admission	2.63 (1.24–5.57)	0.011	0.97	+13
Total bilirubin (per mg/dL)	1.08 (1.02–1.14)	0.008	0.08	+1 per mg/dL
APACHE-II (per 1-point increase)	1.10 (1.05–1.17)	<0.001	0.10	+1.5 per point
Pre-CKRT serum creatinine (per mg/dL)	0.85 (0.76–0.96)	0.006	−0.16	−2 per 1 mg/dl
Pre-CKRT body temperature (per 0.1 °C increase)	0.92 (0.89–0.96)	<0.001	−0.08	−1 per 0.1 °C

No evidence of multicollinearity was observed among predictors, with VIF < 10 and tolerance value > 0.20.

### Development of a prediction score for hypothermia during CKRT

The prediction score was constructed from the final multivariable model. Respiratory failure at ICU admission was weighed at +13 points, total bilirubin was assigned +1 point per 1 mg/dL increase, and APACHE-II score was assigned +1.5 points per 1-point APACHE-II score. In contrast, serum creatinine before CKRT initiation was assigned − 2 points per mg/dL increase, and pre-CKRT body temperature was assigned −1 point for every 0.1 °C increase. The weighted components were summed to generate an individual risk score (Table 2).

### Prediction score discrimination, calibration, and internal validation

**Figure 1.**
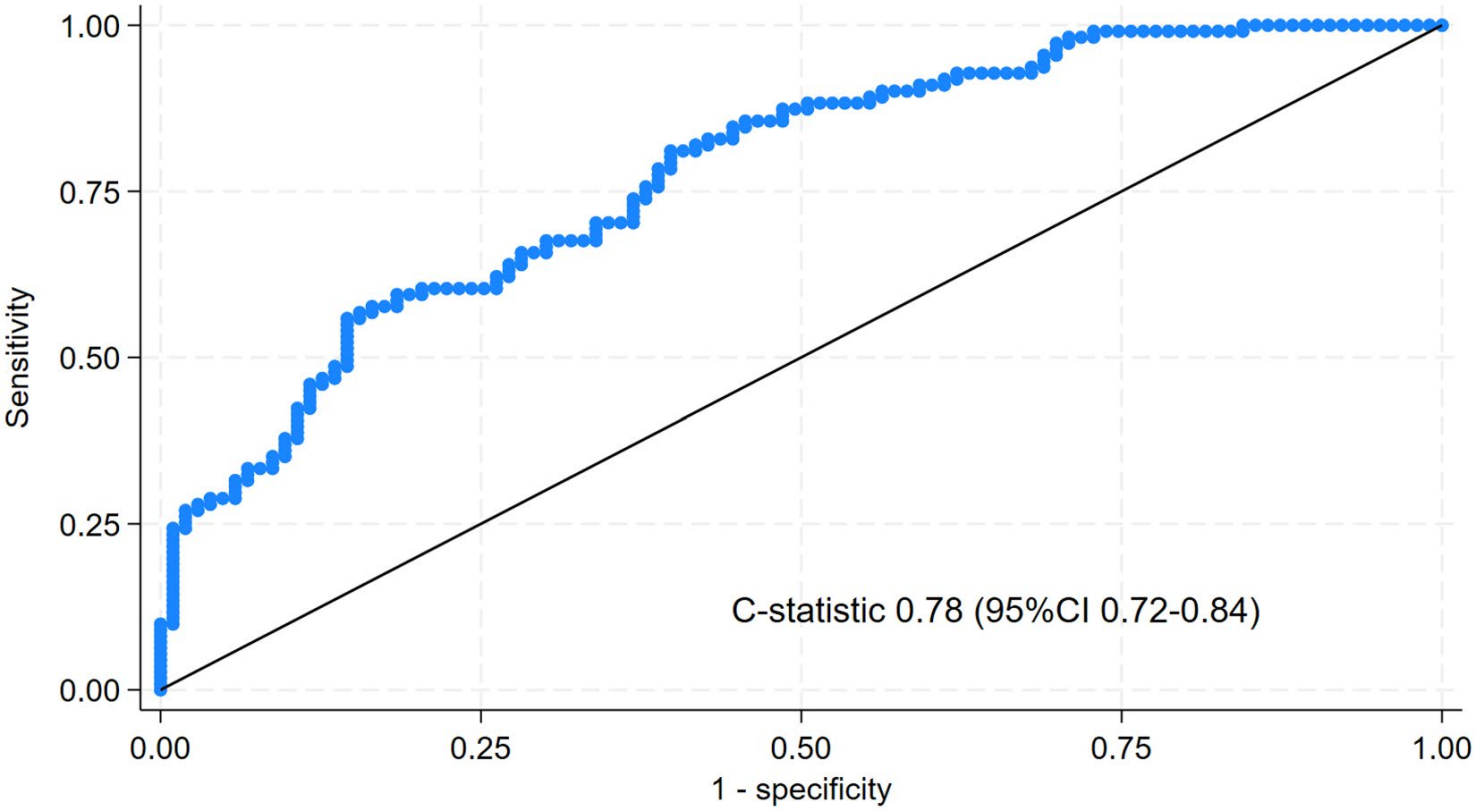
C-statistic of the prediction score for hypothermia during CKRT.

The prediction score demonstrated an acceptable discriminative ability, with a C-statistic of 0.78; 95% CI 0.72–0.84, *p* < 0.001, as shown in Figure 1. After internal validation *via* bootstrapping with 1,000 replications, the model optimism-corrected C-statistic remained 0.78; 95% CI 0.72–0.83, *p* < 0.001.

**Figure 2.**
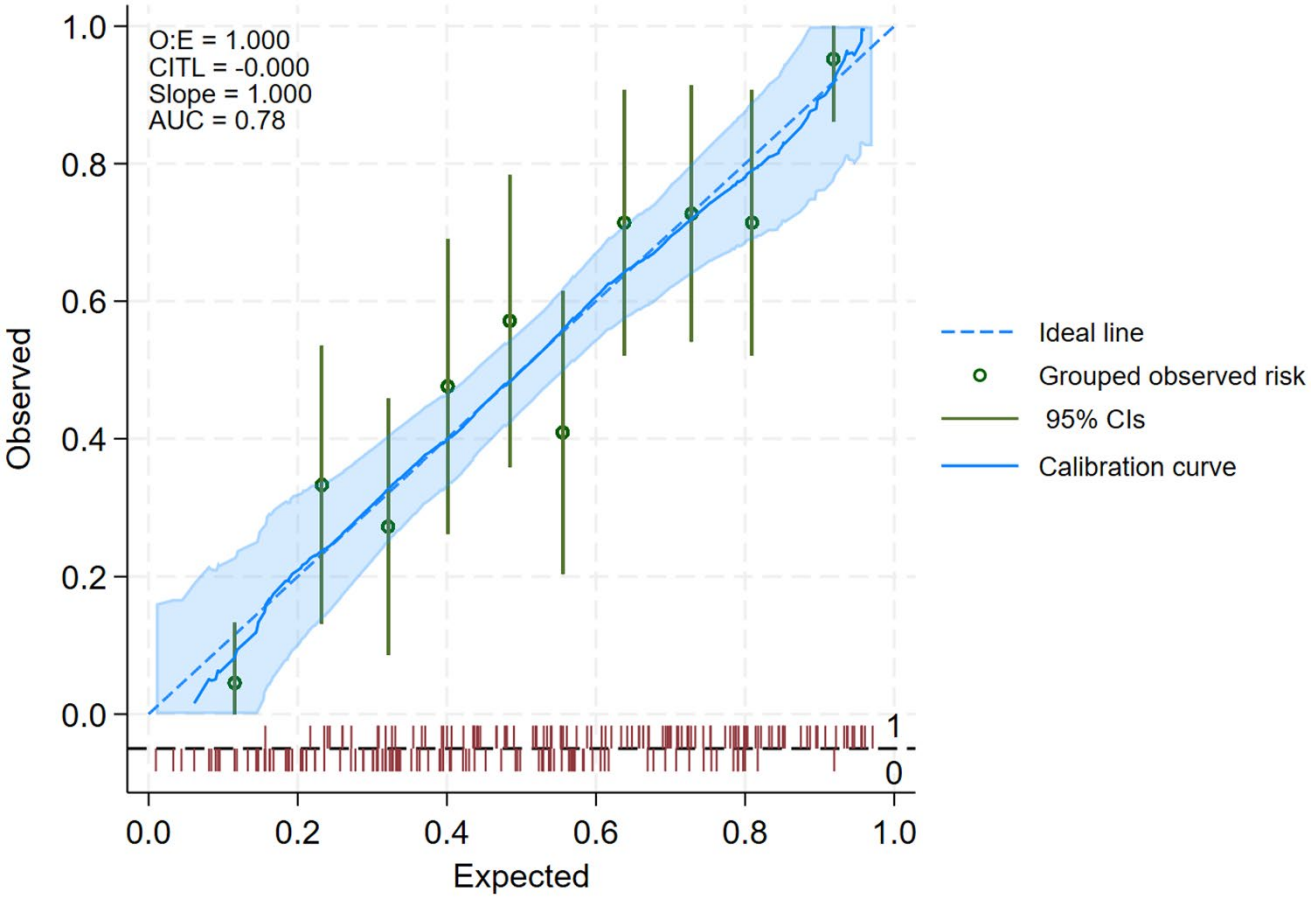
Calibration plot of the hypothermia prediction model during CKRT.

Figure 2 depicts the apparent calibration plot in the derivation cohort, showing close agreement between predicted risk and observed probabilities (O:*E* = 1.000, CITL = 0.000, and calibration slope = 1.000). The HL-GOF was not statistically significant (*p* = 0.470), consistent with the apparent calibration observed in the derivation cohort.

### Optimal cutoff for high risk for hypothermia during CKRT

The optimal cutoff value determined by the Youden index was 18.69. Accordingly, a score of 19.0 identified patients at increased risk of hypothermia during CKRT (Supplementary Table 2). However, a cutoff score of 20.0 was not prespecified but was *post hoc* selected as a clinically pragmatic rounding to provide a more intuitive bedside threshold, offering a balanced sensitivity-specificity tradeoff while maintaining comparable diagnostic performance to adjacent cutoff values (Supplementary Table 2).

At a threshold of 20.0, the sensitivity and specificity were 78 (95% CI 70–86) and 62 (95% CI 52–72), respectively. The corresponding positive and negative likelihood ratios were 2.07 (95% CI 1.59–2.70) and 0.35 (95% CI 0.24–0.51), respectively.

### Risk stratification for hypothermia during CKRT

To refine risk stratification, the prediction scores were categorized into four distinct groups: Group 1 (scores ≤ 0), Group 2 (0–19.9), Group 3 (20.0–39.9), and Group 4 (≥40.0). Hypothermia rates increased across score categories (Table 3).

**Table 3.** Diagnostic performance of hypothermia prediction score by risk strata.

Group	Score range	Hypothermia, *n*	Total, *n*	Event rate (%)	Sensitivity, % (95% CI)	Specificity, % (95% CI)	LR+ (95% CI)	LR- (95% CI)
1	≤0	0	13	0	0 (0–3)	87 (79–93)	n/a	n/a
2	0.01–19.9	24	75	32.0	22 (14–30)	51 (41–61)	0.44 (0.29–0.65)	n/a
3	20.0–39.9	51	83	61.4	46 (36–56)	69 (59–78)	1.48 (1.04–2.10)	0.78 (0.63–0.97)
4	≥40.0	36	43	83.7	32 (24–42)	93 (87–97)	4.77 (2.22–10.24)	0.72 (0.63–0.83)
All		111	214	51.9				

Sensitivity and specificity were calculated for classification at or above each risk stratum. LR+, positive likelihood ratio; LR-, negative likelihood ratio. LR+ was not estimable in Group 1 because no hypothermia events were observed in that stratum. LR- was not reported for Groups 1 and 2 because it is less clinically interpretable in lower-risk strata. LR- values are shown for Groups 3 and 4 as clinically relevant higher-risk thresholds.

No hypothermia events were observed in Group 1. This group showed a specificity of 87% (95% CI 79–93).

In Group 4, 83.7% (36/43 cases) developed hypothermia. This category showed a sensitivity of 32% (95% CI 24–42) and a specificity of 93% (95% CI 87–97), with an associated positive likelihood ratio of 4.77 (95% CI 2.22–10.24) and negative likelihood ratio of 0.72 (95% CI 0.63–0.83) (Table 3).

### Decision curve analysis for clinical use of the hypothermia prediction score

**Figure 3.**
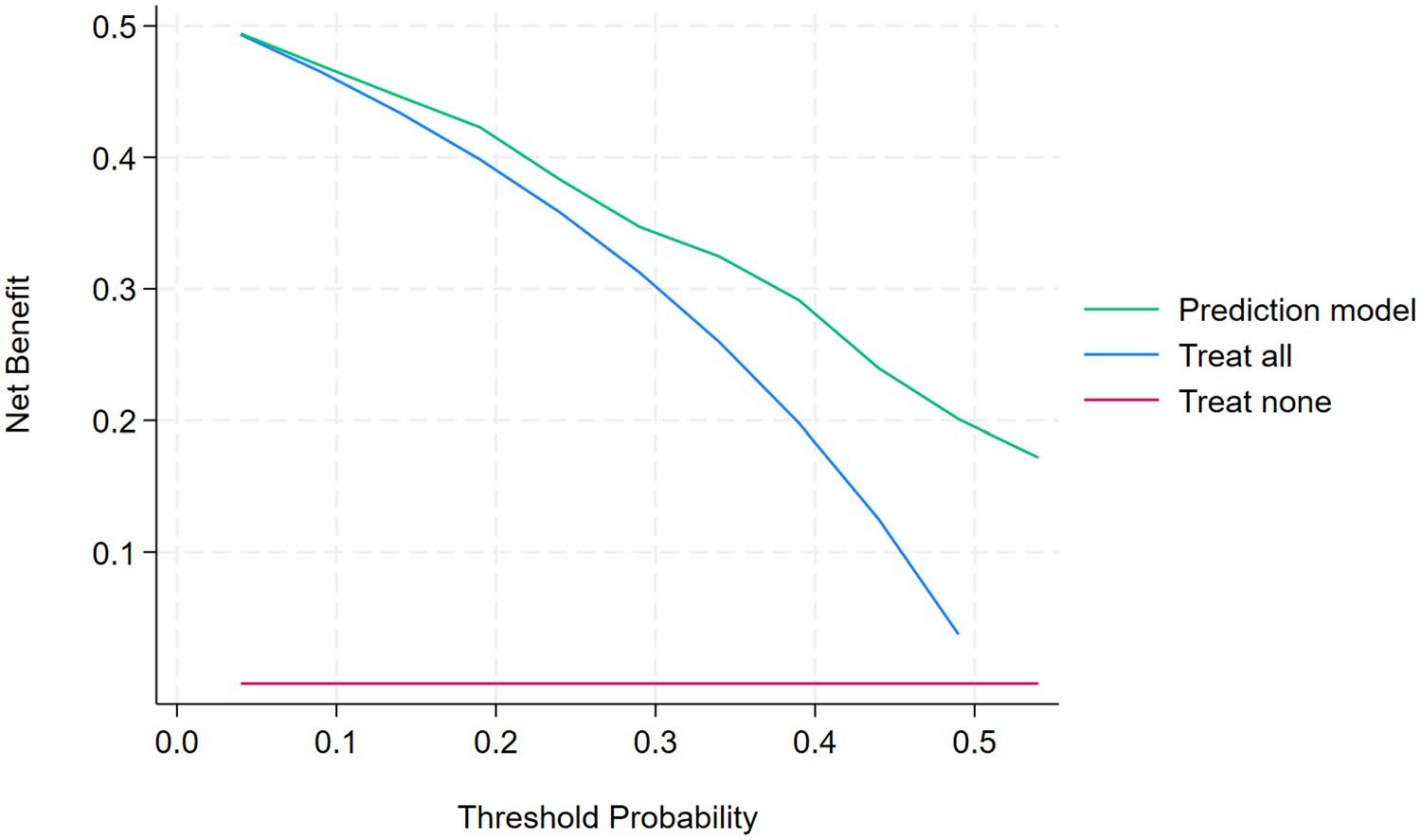
Decision curve analysis of the hypothermia prediction model during CKRT.

The prediction score demonstrated higher net benefit than the treat-all strategy across threshold probabilities of 5%–55% (Figure 3). Net benefit ranged from 0.494 at a 5% threshold to 0.172 at a 55% threshold, while the treat-all strategy declined progressively and became negative at 55% (Supplementary Table 3).

### Post hoc sensitivity analyses excluding patients with end-stage kidney disease

After excluding patients with ESKD, 185 patients remained for analysis. The direction and magnitude of associations for the main predictors were generally consistent with the primary model (Supplementary Table 4). Although respiratory failure at ICU admission and serum creatinine no longer reached conventional statistical significance, the effect estimates were comparable to those observed in the primary analysis. Model discrimination remained acceptable, with a C-statistic of 0.76; 95% CI 0.69–0.83, indicating stable model performance after exclusion of this subgroup.

### Exploratory association between hypothermia and ICU mortality

**Figure 4.**
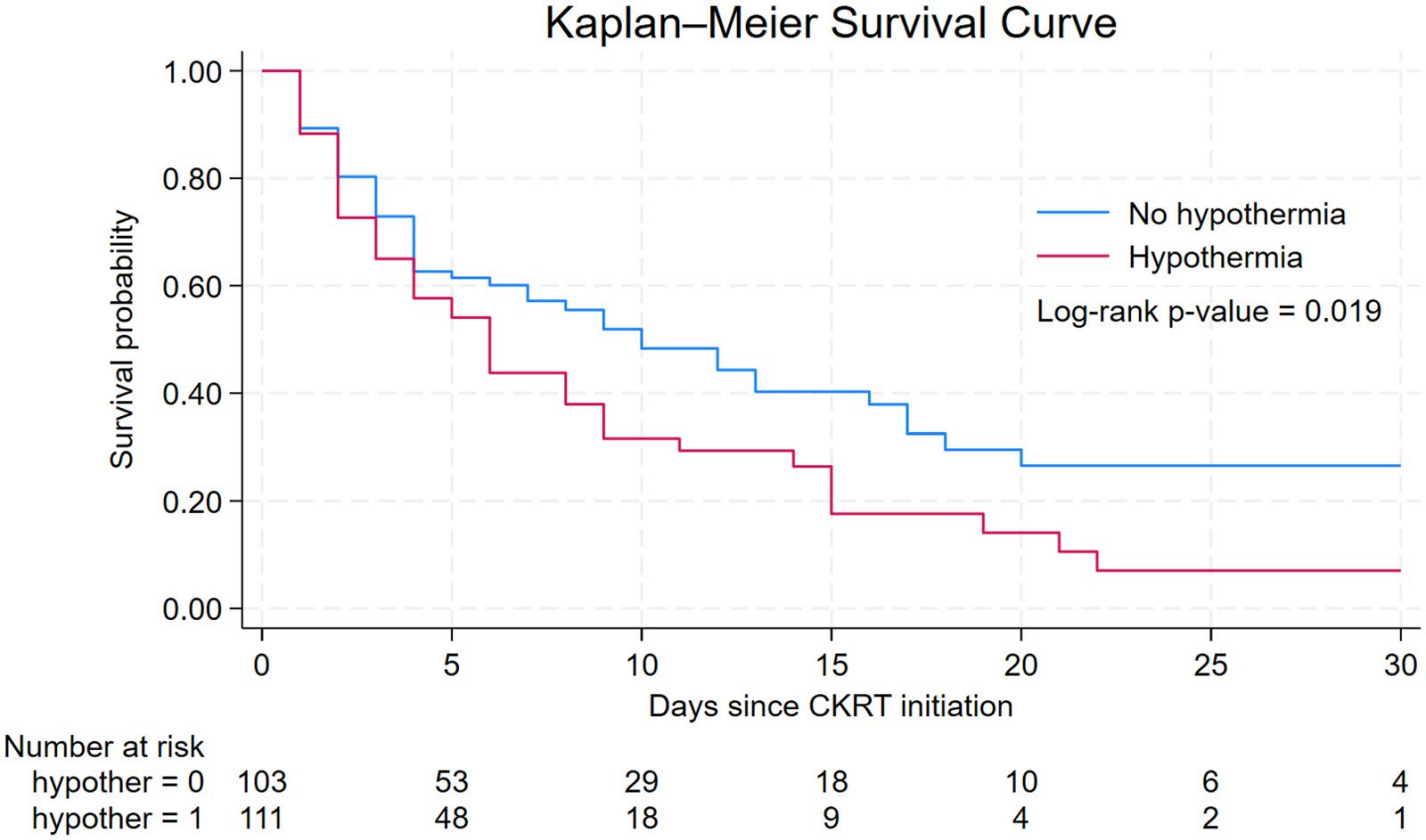
Kaplan–Meier survival curve for ICU mortality in patients with and without CKRT-related hypothermia.

Patients with CKRT-related hypothermia showed significantly lower ICU survival than those without hypothermia (log-rank *p* = 0.019) (Figure 4). In univariable Cox proportional hazards regression analysis, hypothermia was significantly associated with ICU mortality, with a crude HR of 1.50; 95% CI 1.05–2.14, *p* = 0.027. After adjustment for respiratory failure at ICU admission, septic shock, APACHE-II score, and serum lactate, hypothermia remained independently associated with ICU mortality, with an adjusted HR of 1.48; 95% CI 1.01–2.17; *p* = 0.044) (Supplementary Table 5).

## Discussion

Hypothermia occurred in 51.9% of patients within the first 48 h of CKRT initiation, with a median onset of 7 h. In this cohort, predictors identified in the multivariable model reflected underlying organ dysfunction and impaired thermoregulatory capacity. Respiratory failure, elevated total bilirubin, and higher APACHE-II score were independently associated with hypothermia, whereas higher serum creatinine and higher pre-CKRT body temperature were inversely associated with hypothermia risk. These findings suggest that underlying organ dysfunction and illness severity may contribute to thermoregulatory instability during CKRT.

The derived clinical prediction score demonstrated acceptable discrimination (C-statistic of 0.78; 95% CI 0.72–0.84), which remained stable after internal validation. Risk stratification into four categories revealed a clear stepwise increase in hypothermia probability, with progressively higher observed event rates across score groups. This gradient supports the clinical interpretability of the score and enhances its potential application as a bedside tool. By facilitating risk-adapted temperature management, the score enables prioritization of intensive preventive measures for higher-risk patients while maintaining routine monitoring for those at lower risk.

These findings can be interpreted in the context of plausible physiological mechanisms contributing to thermoregulatory instability during CKRT. Respiratory failure was independently linked to hypothermia, possibly due to impaired oxygen delivery and altered vasomotor regulation leading to reduced peripheral heat conservation [10]. In mechanically ventilated patients, deep sedation and neuromuscular blockage (NMB) may further diminish shivering and endogenous heat production, although these data were not captured in our cohort.

Elevated bilirubin, a marker of hepatic dysfunction and illness severity, also showed a significant association with hypothermia [11,12]. Although direct clinical evidence is limited, liver dysfunction may impair thermoregulation, and experimental data suggest a potential hepatic-immune influence on thermogenesis [13]. Consistently, greater illness severity, as indicated by higher APACHE-II score, demonstrated a similar pattern, in line with findings reported by Morsch et al. [2].

In contrast, higher pre-CKRT serum creatinine was inversely associated with hypothermia risk. Although longer or more intensive CKRT exposure could theoretically increase heat loss, this explanation was not supported by our cohort data, in which CKRT duration and prescribed dose were comparable between groups. Because serum creatinine was measured before CKRT initiation, it should not be interpreted as reflecting the delivered CKRT intensity. Rather, serum creatinine may reflect a broader patient phenotype, including skeletal muscle mass and metabolic activity [14]. These characteristics may contribute to greater endogenous heat production and improved thermoregulatory resilience. However, given its dependence on renal function and acute illness [15], this association should be interpreted cautiously.

Baseline body temperature proved to be one of the strongest predictors. Prior work demonstrated that each 1 °C increment substantially lowered the risk of hypothermia [16], and similarly in our cohort, each 0.1 °C increase in pre-CKRT body temperature independently reduced the likelihood of hypothermia (OR 0.92; 95% CI 0.89–0.96; *p* < 0.001).

Hypothermia in critical illness may result from increased heat loss, reduced heat production, or impaired thermoregulation [2,3,6,17,18]. Heat loss commonly occurs due to environmental exposure or medical interventions such as extracorporeal circulation [2,3] and infusion of cooled fluids [18]. Reduced heat production is typically seen in elderly individuals or those with inadequate nutritional or metabolic reserves [6]. Impaired thermoregulation may arise from peripheral vascular dysfunction, neuropathy, or cerebrovascular injury, all of which can compromise central temperature control [6,18].

In the CKRT setting, hypothermia is primarily attributed to heat loss during extracorporeal circulation, as blood is exposed to ambient temperature within the CKRT circuit [2,19]. This mechanism likely explains the high incidence observed in our cohort, despite the use of modern CKRT machines equipped with integrated blood-warming systems.

Previous studies have reported varying incidences of hypothermia during CKRT. Yagi et al. reported hypothermia in 38% of CKRT sessions [4], with higher incidence observed in CVVHD (48%) and CVVH (40%). However, that study was conducted in 1998, and the specific CKRT platforms were not described. Given the substantial technological evolution in CKRT systems and thermal management practices over the past two decades, differences in equipment design and institutional protocols may partly explain discrepancies with contemporary findings.

In a recent study, Morsch et al. reported an overall hypothermia incidence of 52.7%, with a higher rate in CVVHDF (59.2%) than CVVHD (40.8%, *p* = 0.003) [2]. In that cohort, CVVHD was performed using a Diapact system (priming volume ∼ 300 mL), whereas CVVHDF was delivered using a Prismaflex platform (priming volume ∼ 150 mL). Despite the smaller circuit volume in CVVHDF, neither priming volume nor replacement fluid flow rate was significantly associated with hypothermia, suggesting that platform-specific heating mechanisms may have contributed to modality-related differences in thermal loss.

In contrast, our study did not demonstrate a significant difference in hypothermia incidence among CKRT modalities (*p* = 0.45). At our center, CKRT was predominantly performed using the Aquarius platform (96% of treatments), with a priming volume of approximately 250–300 mL. The relative uniformity of CKRT systems and institutional thermal management protocols in our setting may have attenuated modality-specific thermal differences, thereby limiting observed variability across modalities in our cohort.

Hypothermia in critically ill patients has been associated with increased ICU mortality, potentially through mechanisms such as immune suppression, arrhythmia, hemodynamic instability, and reduced responsiveness to catecholamines [5,20–23]. In our study, although crude ICU mortality proportions did not show a statistically significant between-group difference (64.9% vs. 53.4%, *p* = 0.10), exploratory time-to-event analysis demonstrated that hypothermia was independently associated with increased ICU mortality in the overall cohort (adjusted HR 1.48; 95% CI 1.01–2.17; *p* = 0.044). This finding aligns with prior studies reporting mortality rates of 58.2%–71.0% among hypothermic CKRT patients [2–4]. Furthermore, studies in general ICU populations have shown increased mortality even at *a* < 36 °C threshold [24,25], highlighting the clinical significance of mild temperature reductions.

### Application

This study presents a clinically applicable hypothermia prediction score derived from routinely collected bedside vital signs and laboratory parameters. The use of pre-CKRT data enhances its feasibility for bedside implementation and early risk stratification. By identifying patients at increased risk before hypothermia occurs, the model may support proactive temperature surveillance and timely preventive measures during CKRT. Additionally, the incorporation of variables reflecting organ dysfunction and admission diagnoses provided further determinants of thermoregulatory instability in critically ill patients. Future studies should compare this clinically interpretable score with alternative modeling strategies, including penalized regression approaches such as LASSO, to evaluate whether discrimination, calibration, and generalizability can be further improved.

### Strengths

This study introduces a clinically practical hypothermia prediction score based on readily available vital signs and laboratory parameters. The use of routine admission data allows for efficient risk stratification at the bedside. Moreover, the inclusion of diverse clinical variables, including organ dysfunction and admission diagnoses, provides insight into previously underexplored risk factors associated with hypothermia.

### Limitations

Several limitations merit consideration. First, temperature was measured *via* the axillary route rather than core temperature monitoring, which may have resulted in misclassification. However, the consistent measurement across all patients likely minimized systematic bias. Because the same axillary temperature measurement method was used uniformly across all patients, any resulting outcome misclassification would likely be non-differential and may attenuate observed associations rather than exaggerate model performance. Moreover, ambient ICU temperature was not formally recorded. Nonetheless, all patients were managed in a climate-controlled environment maintained within the standard range for critical care settings (approximately 20 °C–24 °C), which likely limited substantial environmental variability. Importantly, these findings highlight hypothermia as a frequent complication during CKRT in our setting and may support the future implementation of more structured temperature monitoring and preventive management protocols.

Second, the cutoff value of 20 was selected to enhance clinical practicality. Although slightly lower thresholds provided marginally higher sensitivity, they did not offer meaningful improvement in overall performance. Different thresholds may perform differently in external populations and therefore require further validation.

Third, certain potentially relevant variables, including detailed sedation practices, NMB use, nutritional status, metabolic deficiencies, and hormonal disturbances, were not systematically collected, precluding more detailed mechanistic exploration. Fourth, although the initial sample size was based on the traditional events-per-variable rule, this approach may be insufficient under contemporary methodological standards for prediction model development. A contemporary sample size sensitivity analysis based on the Riley framework suggested that a larger cohort (approximately 384–434 patients depending on assumed model complexity) would improve precision of baseline risk estimation and further reduce potential model overfitting.

Fifth, the model demonstrated close apparent calibration in the derivation cohort. However, these findings reflect model performance within the development dataset and should therefore be interpreted cautiously. External validation is required to confirm calibration performance in independent populations before stronger conclusions regarding model transportability can be drawn.

Finally, as this study was conducted in a medical ICU, the findings may not be fully generalized to surgical, cardiological, and mixed ICU settings, where factors such as open abdominal surgery, cardiogenic shock, or extensive burn injuries may further influence thermoregulation. Therefore, external validation in these independent ICU populations is required before clinical adoption to assess model generalizability across different case mixes and thermoregulatory risk profiles.

## Conclusions

Hypothermia is common during CKRT in critically ill patients and reflects both treatment-related heat loss and underlying illness severity. Respiratory failure, elevated total bilirubin, and higher APACHE-II scores were independently associated with its development, whereas higher serum creatinine and pre-CKRT body temperature were inversely associated with hypothermia risk. These factors informed the derivation of a clinically applicable prediction score with acceptable discrimination. Hypothermia was independently associated with increased ICU mortality, underscoring its clinical significance and the potential value of early risk identification to support proactive temperature management during CKRT.

## Supplementary Material

Supplementary Material Hypothermia.docx

## Data Availability

The data that support the findings of this study are available from the corresponding author, [KT], upon reasonable request.

## Generative artificial intelligence (AI)

The authors used a generative artificial intelligence tool (ChatGPT, OpenAI) to assist in improving language clarity, grammar, and manuscript structure. All AI-generated contents were carefully reviewed, revised, and verified by the authors. The authors take full responsibility for the accuracy, integrity, and originality of the work.
