# Supplementary material for: Development of a predictive score for hypothermia risk during continuous kidney replacement therapy in critically ill patients
Source: Ren Fail. 2026 Jun 1;48(1):2676486. doi: 10.1080/0886022X.2026.2676486 (PMC13231807; doi:10.1080/0886022X.2026.2676486)
Supplement: Supplementary Material Hypothermia.docx [file IRNF_A_2676486_SM1329.docx]

**Development of a Predictive Score for Hypothermia Risk During Continuous Kidney Replacement Therapy in Critically Ill Patients**

**Supplementary Material**

Supplementary Table 1 Risk factors for hypothermia during CKRT.

Supplementary Table 2 Optimal cut-off for high risk for hypothermia during CKRT.

Supplementary Table 3 Decision curve analysis of the hypothermia prediction score during CKRT.

Supplementary Table 4 Post hoc sensitivity analyses excluding patients with ESKD (n=185).

Supplementary Table 5 Adjusted HR for risk of hypothermia and ICU mortality.

Supplement Table 1 Risk factors for hypothermia during CKRT.

| Variables | Univariable analysis | | Multivariable analysis | |
| --- | --- | --- | --- | --- |
|  | Odds Ratio (95% CI) | p-value | Odds Ratio (95% CI) | p-value |
| Demographic |  |  |  |  |
| Age (years) | 1.03 (1.01–1.04) | 0.002 | – | – |
| Female, n (%) | 1.22 (0.70–2.11) | 0.49 | – | – |
| Body Mass Index (kg/m^2^) | 0.91 (0.85–0.98) | 0.009 | – | – |
| Pre-existing Comorbidities, n (%) |  |  |  |  |
| Hypertension | 1.10 (0.64–1.89) | 0.72 | – | – |
| Diabetes mellitus | 0.76 (0.43–1.32) | 0.32 | – | – |
| Chronic kidney disease | 0.91 (0.48–1.71) | 0.76 | – | – |
| Malignancy | 0.96 (0.50–1.83) | 0.90 | – | – |
| Dyslipidemia | 0.91 (0.47–1.77) | 0.78 | – | – |
| Chronic liver disease | 1.16 (0.59–2.27) | 0.68 | – | – |
| Arrhythmia | 1.05 (0.51–2.19) | 0.89 | – | – |
| Coronary artery disease | 1.92 (0.85–4.35) | 0.12 | – | – |
| End stage kidney disease | 0.52 (0.23–1.16) | 0.11 | – | – |
| Other comorbidities | 1.05 (0.61–1.81) | 0.86 | – | – |
| Diagnosis at ICU Admission, n (%) |  |  |  |  |
| Septic shock | 1.10 (0.56–2.16) | 0.79 | – | – |
| Source of infection, n (%) |  |  |  |  |
| Respiratory infection | 1.09 (0.64–1.87) | 0.75 | – | – |
| Urosepsis | 0.70 (0.34–1.44) | 0.33 | – | – |
| Gastrointestinal tract infection | 0.77 (0.34–1.75) | 0.54 | – | – |
| Organ Failure at ICU Admission, n (%) |  |  |  |  |
| Cardiovascular | 0.70 (0.30–1.62) | 0.40 | – | – |
| Renal | 0.63 (0.27–1.49) | 0.29 | – | – |
| Respiratory | 2.53 (1.32–4.86) | 0.005 | 2.63 (1.25–5.57) | 0.011 |
| Gastrointestinal | 0.19 (0.04–0.91) | 0.038 | – | – |
| Others | 0.48 (0.17–1.34) | 0.16 | – | – |
| Vital signs at CRRT initiation |  |  |  |  |
| Pre-CRRT body temperature (℃) | 0.54 (0.40–0.72) | <0.001 | 0.46 (0.33–0.64) | <0.001 |
| Mean arterial pressure (mm Hg) | 0.99 (0.97–1.02) | 0.58 | – | – |
| Heart rate (bpm) | 1.05 (1.00–1.10) | 0.06 | – | – |
| Respiratory rate (bpm) | 0.99 (0.98–1.00) | 0.045 | – | – |
| Oxygen saturation (%) | 0.97 (0.93–1.02) | 0.26 | – | – |
| Glasgow Coma Score | 0.91 (0.84–0.98) | 0.018 | – | – |

Supplement Table 1 Risk factors for hypothermia during CKRT (cont).

| Variables | Univariable analysis  Odds Ratio (95% CI) | p-value | Multivariable analysis Odds Ratio (95% CI) | p-value |
| --- | --- | --- | --- | --- |
| Laboratory Before CRRT Initiation |  |  |  |  |
| Hemoglobin (g/dl) | 0.94 (0.84–1.05) | 0.27 | – | – |
| White blood cell counts (10^3^ cells/ mm^3^) | 1.00 (0.99–1.00) | 0.45 | – | – |
| Platelet count (10^3^ cells/ mm^3^) | 1.00 (0.99–1.00) | 0.79 | – | – |
| Total bilirubin (mg/dL) | 1.05 (1.00–1.11) | 0.038 | 1.08 (1.02–1.14) | 0.008 |
| Blood urea nitrogen (mg/dL) | 1.00 (0.99–1.01) | 0.49 | – | – |
| Baseline serum creatinine (mg/dL) | 0.87 (0.76–1.00) | 0.05 | – | – |
| Serum creatinine (mg/dL) | 0.85 (0.77–0.94) | 0.002 | 0.85 (0.76–0.96) | 0.006 |
| APACHE-II score | 1.08 (1.03–1.13) | 0.001 | 1.105 (1.05–1.17) | <0.001 |
| CRRT Indication, n (%) |  |  |  |  |
| Severe AKI | 1.08 (0.62–1.88) | 0.78 | – | – |
| Electrolyte imbalance | 0.61 (0.35–1.08) | 0.09 | – | – |
| Volume overload | 3.67 (0.99–13.54) | 0.05 | – | – |
| Uremic complication | 0.92 (0.31–2.73) | 0.89 | – | – |

Among organ failures at ICU admission, only respiratory failure remained independently associated with hypothermia in the multivariable model. Although gastrointestinal failure was statistically significant in the initial multivariable model, it was observed in only 11 patients (5%). Given the risk of sparse data bias and model instability, this variable was excluded from the final prediction model to enhance robustness and clinical applicability. Therefore, the final model was prioritized predictors with sufficient prevalence and biological plausibility.

Supplementary Table 2 Optimal cut-off for high risk for hypothermia during CKRT.

| Cut-off point | Sensitivity,  % (95% CI) | Specificity,  % (95% CI) | LR+  (95% CI) | LR-  (95% CI) |
| --- | --- | --- | --- | --- |
| 18.5 | 84 (76–90) | 58 (48–68) | 2.01 (1.57–2.56) | 0.28 (0.18–0.44) |
| 19.0 | 82 (74–89) | 59 (49–69) | 2.01 (1.57–2.58) | 0.30 (0.20–0.47) |
| 19.5 | 80 (72–87) | 60 (50–70) | 2.01 (1.56–2.60) | 0.33 (0.22–0.49) |
| 20.0 | 78 (70–86) | 62 (52–72) | 2.07 (1.59–2.70) | 0.35 (0.24–0.51) |
| 20.5 | 77 (68–84) | 62 (52–72) | 2.02 (1.55–2.64) | 0.38 (0.26–0.54) |

Abbreviation: LR+, positive likelihood ratio and LR-, negative likelihood ratio.

Supplementary Table 3 Decision curve analysis of the hypothermia prediction score during CKRT.

| Threshold probability for hypothermia during CKRT (%) | Treat All | Net Benefit | Reduced number of overtreatments per 100 patients |
| --- | --- | --- | --- |
| 5 | 0.493 | 0.494 | 1.402 |
| 10 | 0.465 | 0.470 | 4.201 |
| 15 | 0.434 | 0.446 | 7.009 |
| 20 | 0.398 | 0.423 | 9.813 |
| 25 | 0.358 | 0.383 | 7.477 |
| 30 | 0.312 | 0.347 | 8.100 |
| 35 | 0.260 | 0.325 | 12.083 |
| 40 | 0.198 | 0.291 | 14.019 |
| 45 | 0.125 | 0.240 | 14.019 |
| 50 | 0.037 | 0.201 | 16.355 |
| 55 | -0.070 | 0.172 | 19.754 |

Supplementary Table 4 Post hoc sensitivity analyses excluding patients with ESKD (n = 185).

| Predictors | OR (95% CI) | p-value | Coefficient |
| --- | --- | --- | --- |
| Respiratory failure at ICU admission | 2.13 (0.96–4.2) | 0.063 | 0.76 |
| Total bilirubin (per mg/dl) | 1.08 (1.02–1.14) | 0.010 | 0.08 |
| APACHE-II (per 1-point increase) | 1.11 (1.05–1.18) | <0.001 | 0.11 |
| Serum creatinine (per mg/dL) | 0.87 (0.76–1.01) | 0.062 | -0.14 |
| Pre-CKRT body temperature (per 0.1℃ increase) | 0.93 (0.89–0.96) | <0.001 | -0.08 |

Supplementary Table 5 Adjusted HR for risk of hypothermia and ICU mortality.

| Variables | Hazard ratio (95% CI) | p-value |
| --- | --- | --- |
| Hypothermia | 1.48 (1.01–2.17) | 0.044 |
| Respiratory failure | 0.79 (0.51–1.22) | 0.70 |
| Septic shock | 1.10 (0.68–1.77) | 0.70 |
| APACHE-II | 1.01 (0.98–1.04) | 0.59 |
| Serum lactate | 1.01 (1.002–1.02) | 0.022 |
